# Mechanical ventilation in aneurysmal subarachnoid hemorrhage: systematic review and recommendations

**DOI:** 10.1186/s13054-020-03269-8

**Published:** 2020-09-24

**Authors:** James E. Towner, Redi Rahmani, Christopher G. Zammit, Imad R. Khan, David A. Paul, Tarun Bhalla, Debra E. Roberts

**Affiliations:** 1 Department of Neurosurgery, University of Rochester Medical Center, 601 Elmwood Ave, Rochester, NY 14642 USA; 2 Division of Neurocritical Care, Department of Neurology, University of Rochester Medical Center, 601 Elmwood Ave, Rochester, NY 14642 USA; 3 Division of Pulmonary Diseases and Critical Care, Department of Medicine, University of Rochester Medical Center, 601 Elmwood Ave, Rochester, NY 14642 USA; 4 TriHealth Critical Care, 10506 Montgomery Road, Suite 301, Cincinnatir, OH 45242 USA; 5 Department of Imaging Sciences, University of Rochester Medical Center, 601 Elmwood Ave, Box 670, Rochester, NY 14642 USA; 6 Department of Neurology, University of Rochester Medical Center, 601 Elmwood Ave, Rochester, NY 14642 USA; 7 Department of Anesthesiology and Perioperative Medicine, University of Rochester Medical Center, 601 Elmwood Ave, Rochester, NY 14642 USA

**Keywords:** Mechanical ventilation, Aneurysm, Subarachnoid hemorrhage, APRV, Pressure control, Volume control

## Abstract

**Objective:**

Mechanical ventilation (MV) has a complex interplay with the pathophysiology of aneurysmal subarachnoid hemorrhage (aSAH). We aim to provide a review of the physiology of MV in patients with aSAH, give recommendations based on a systematic review of the literature, and highlight areas that still need investigation.

**Data sources:**

PubMed was queried for publications with the Medical Subject Headings (MeSH) terms “mechanical ventilation” and “aneurysmal subarachnoid hemorrhage” published between January 1, 1990, and March 1, 2020. Bibliographies of returned articles were reviewed for additional publications of interest.

**Study selection:**

Study inclusion criteria included English language manuscripts with the study population being aSAH patients and the exposure being MV. Eligible studies included randomized controlled trials, observational trials, retrospective trials, case-control studies, case reports, or physiologic studies. Topics and articles excluded included review articles, pediatric populations, non-aneurysmal etiologies of subarachnoid hemorrhage, mycotic and traumatic subarachnoid hemorrhage, and articles regarding tracheostomies.

**Data extraction:**

Articles were reviewed by one team member, and interpretation was verified by a second team member.

**Data synthesis:**

Thirty-one articles met the inclusion criteria for this review.

**Conclusions:**

We make recommendations on oxygenation, hypercapnia, PEEP, APRV, ARDS, and intracranial pressure monitoring.

## Background

Aneurysmal subarachnoid hemorrhage (aSAH) occurs in 10–15 patients per 100,000 annually and represents 10% of all strokes in the USA [1, 2]. Among ischemic, hemorrhagic, and aneurysmal strokes, aSAH is associated with the highest risk of requiring mechanical ventilation (MV) (RR, 3.9; 95% CI 3.8–4.0), with 38.5–65% of all aSAH patients requiring MV [3–6]. Pathologic processes such as neurogenic pulmonary edema (NPE), occurring in up to 20% of aSAH patients, illustrate the interconnectedness of the central nervous and pulmonary systems [7, 8]. Modulating oxygenation and ventilation can be particularly challenging due to a large percentage of aSAH patients suffering from one or more pulmonary complications, including pneumonia (22%) and pulmonary edema (23%) [9]. Additionally, 18–50% of patients with aSAH experience acute respiratory distress syndrome (ARDS) [10–12].

**Fig. 1:**
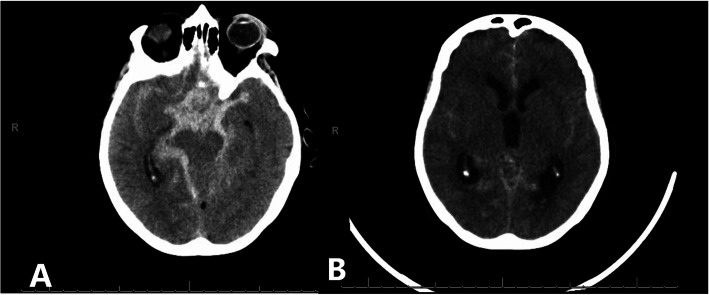
Head CT showing the unique challenges of an aneurysmal subarachnoid hemorrhage patient. **a** There is a significant amount of basilar cistern subarachnoid hemorrhage placing the patient at high risk for delayed cerebral ischemia. **b** There is prominent hydrocephalus needing CSF diversion to lower intracranial pressure

For a pathology that so frequently requires MV, intensivists and neurosurgeons alike are faced with questions regarding optimal management without an abundance of guiding evidence. Unlike other forms of stroke, the clinician must be increasingly cognizant of brain oxygen delivery and perfusion when considering the potential for delayed cerebral ischemia (DCI) and increased intracranial pressure from hydrocephalus (Fig. 1). Herein, we performed a systematic review of all aneurysmal subarachnoid studies with concomitant mechanical ventilation to review factors of ventilation that may be involved with brain-lung physiology. We outline the unique pulmonary pathophysiology of such patients, common conditions encountered, and how each element of mechanical ventilation can impact the complex disease processes of patients with aSAH. In addition, we make recommendations based on existing data and highlight gaps in knowledge for future research.

## Methods

A PubMed database search was formulated based on the PICO (Participant, Intervention, Comparison, and Outcome) framework. The following Medical Subject Headings (MeSH) terms were used to define the participant and the intervention, respectively: “aneurysmal subarachnoid hemorrhage” and “mechanical ventilation.” Search parameters were limited to English language studies published between January 1, 1990, and March 1, 2020. The search was completed on March 1, 2018, and updated on March 8, 2020. Study inclusion criteria included English language manuscripts with the study population being aneurysmal subarachnoid hemorrhage and the exposure or intervention being mechanical ventilation. Eligible studies could include randomized controlled trials, observational trials, retrospective trials, case-control studies, case reports, or physiologic studies. We specifically assessed mechanical ventilation variables, brain physiologic measurements, and outcomes. We excluded topic review articles and articles concerning pediatric populations, non-aneurysmal etiologies of subarachnoid hemorrhage, mycotic and traumatic subarachnoid hemorrhage, and tracheostomies. The citations of the articles returned from the PubMed database search were examined, and any articles that appeared to pertain to our topic were reviewed and included, if appropriate. Bias was assessed individually given the type of study reported. Results found were not similar enough for combination or further statistical analysis.

All articles returned in the search were reviewed. Manuscripts’ abstracts were reviewed by one team member (a neurosurgery resident), and interpretation was verified by a second team member (a critical care physician). Those passing abstract review then underwent full review by two team members in the same manner. 

**Fig. 2:**
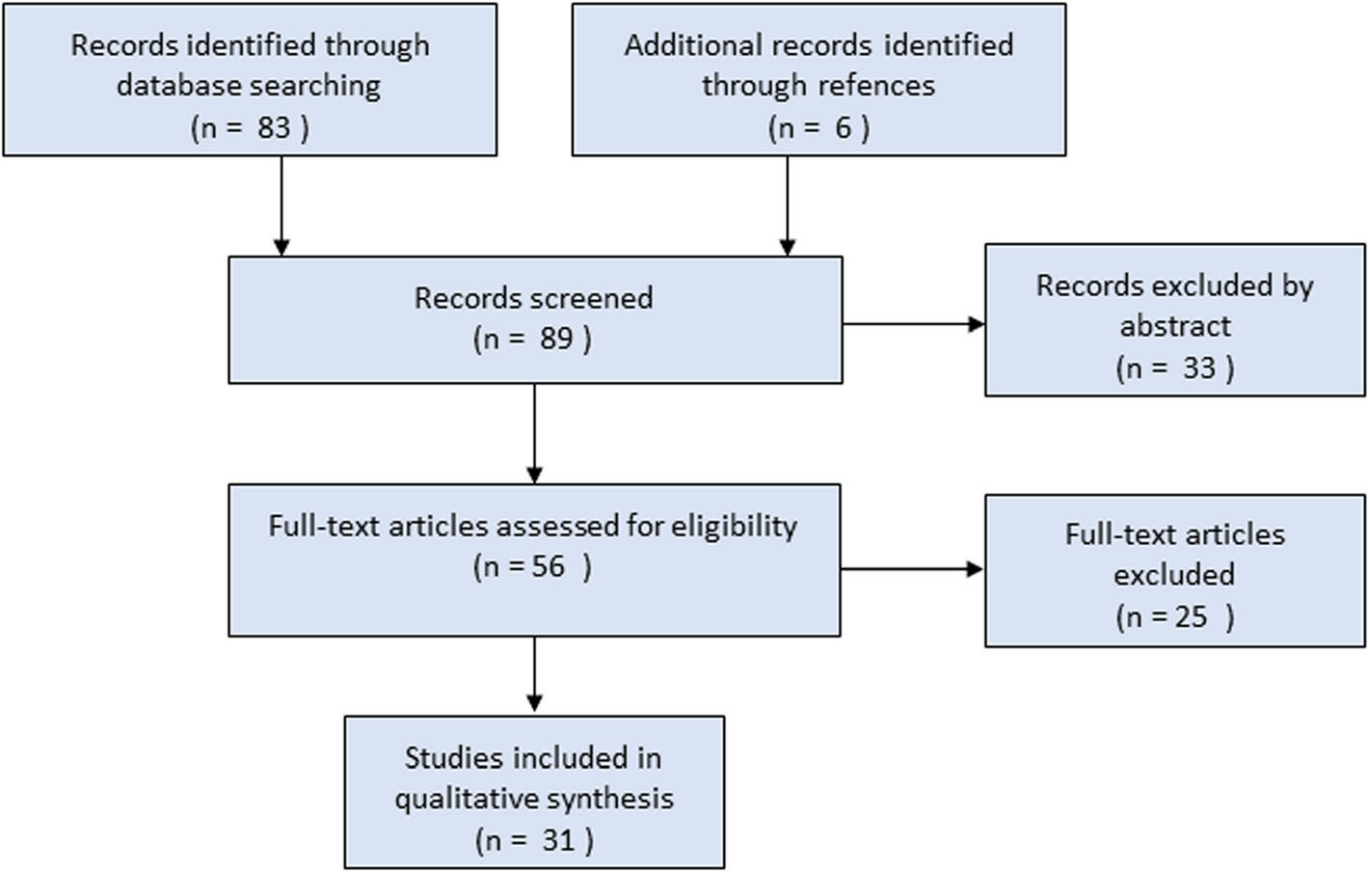
PRISMA flow sheet for systematic review

Our search yielded 83 manuscripts. Of these, 58 articles were ultimately excluded. An additional six manuscripts pertaining to MV in aSAH were identified upon examination of the references of the reviewed articles. Figure 2 is a flow diagram adapted from PRISMA (Preferred Reporting Items for Systematic Reviews and Meta-Analyses) [13]. The total number of articles relevant to this review was 31.

## Oxygen delivery and carbon dioxide clearance

### Oxygenation

Blood oxygen (O_2_) and, to a greater degree, carbon dioxide (CO_2_) are important variables to consider in the management of aSAH patients. Maintaining O_2_ delivery is critically important to avoid brain ischemia. MV must strike a balance between oxygenating blood and maintaining cardiac output.

While there is an accepted goal to avoid hypoxemia in any critically ill patient, the effect of hyperoxemia in aSAH is less well understood. A retrospective, multi-institutional database study of aSAH patients mechanically ventilated for a minimum of 24 h found no relationship between PaO_2_ level in the first 24 h of care, including moderate hyperoxemia (≥ 150 mmHg), and patient outcome [14].

### Carbon dioxide

CO_2_ is an important and powerful vasomodulator, potentially having a dramatic effect on the delivery of blood to the brain in patients with intact cerebral autoregulation [15]. Studies, both in humans and animal models, have found functioning cerebrovascular reactivity to CO_2_ following aSAH [14, 16]. A retrospective, single-institution review of 102 aSAH patients found hypocapnia (defined as a partial pressure of CO_2_ (PaCO_2_) < 35) to be independently associated with unfavorable outcome (defined as Glasgow outcome scale (GOS) < 4) and DCI, but not mortality [17]. Conversely, PaCO_2_ levels above 37.5 mmHg in the first 24 h of care have been associated with a decreased risk of unfavorable outcome (defined as GOS of 1–3), suggesting that permissive mild hypercapnia may be beneficial in aSAH patients [18]. Westermaier et al. conducted a phase 1 clinical trial of controlled transient hypercapnia in aSAH. The authors examined six high-grade (Hunt and Hess (HH) 3–5 and Fisher grade (FG) 3) aSAH patients with multimodality monitoring, including intracerebral thermodilution probes, external ventricular drains (EVD), and near-infrared spectroscopy [19]. They found that PaCO_2_ levels of 30, 40, 50, and 60 mmHg resulted in baseline cerebral blood flow (CBF) changes of 79%, 98%, 124%, and 143%, respectively [19]. The cerebral tissue oxygenation for PaCO_2_ levels of 30, 40, 50, and 60 mmHg changed from baseline by 93%, 98%, 104%, and 111%, respectively [19]. Intracranial pressure (ICP) was not clinically affected by changes in PaCO_2_, but the average amount of cerebrospinal fluid (CSF) drained increased with increasing PaCO_2_ [19]. They experienced no rebound perfusion deficit upon return to baseline ventilator settings [19]. While the ICPs were not elevated in these patients, it was likely only due to the elevated CSF drainage and thus this may be an unsafe maneuver in patients without an EVD. Despite some investigation into permissive hypercapnia as a therapy, there is still significant work to be done in regard to safety and efficacy given that some believe that brain-injured patients should avoid hypercapnia due to the risk of increased ICP from the rise in CBF [20].

### Recommendations

In summary, hypoxemia should be avoided in aSAH patients as with any critically ill patient. While hyperoxemia does not have strong clinical evidence of causing further brain injury, advanced intracranial monitoring to measure brain tissue oxygen pressure (PbtO_2_) can be used when titrating the fraction of inspired oxygen. Permissive hypercapnia is likely well tolerated in these patients, but it may safest to do so with intracranial pressure monitoring (ICPM) in place. Hypocapnia should generally be avoided unless there is an acute rise in ICP since it may incite ischemia.

## Acute respiratory distress syndrome in aSAH

### PEEP and intracranial pressure

ARDS is not uncommon in aSAH. A retrospective, single-institution cohort study of 620 aSAH patients found that 27% had a PaO2:FiO_2_ (fraction of inspired oxygen) ratio of ≤ 300 with 18% having a ratio ≤ 200 [11]. The diagnosis of lung injury occurred a median of 3 days from admission [11]. They found severity of illness, clinical grade of hemorrhage, red blood cell transfusions, and severe sepsis to be independently associated with developing ARDS [11]. Higher tidal volumes were not found to be associated with the subsequent development of ARDS [11]. ARDS was independently associated with mortality and longer hospital lengths of stay [11].

Another retrospective, single-institution observational study found that the development of lung injury was correlated with HH score (*p* < 0.001), with 30.4% of HH4 and 35.5% of HH5 patients experiencing severe lung injury (defined by the authors as a PaO_2_ to FiO_2_ ratio of ≤ 200) [6]. An additional retrospective, observational study of 62 patients with aSAH requiring MV found 50% of their cohort developed ARDS. Forty-five percent of the patients were diagnosed with ARDS on the first day of MV—suggesting hypoxia, not solely the need for airway protection, may contribute to the requirement for MV [12].

The hypothesized pathophysiology leading to the development of lung injury in aSAH patients is a “double-hit” model, with the first hit being an adrenergic surge and systemic inflammation incited by acute neurologic injury and the second from non-neurological stressors, such as infections, transfusions, and MV [21–23]. The best evidence for MV strategies to improve survival in ARDS involves lung-protective ventilation parameters described in an ARDS Network trial (ARDSNet) which includes tidal volumes of 6–8 mL/kg of predicted body weight to achieve a plateau pressure ≤ 30 cmH_2_O [24]. However, it is important to recognize that patients with elevated ICPs were excluded from the ARDSNet trial, which likely excluded many aSAH patients. Such an exclusion may limit the generalizability of this ventilation strategy to patients with aSAH.

When examining the utilization rates of the ARDSNet lung-protective ventilator strategies in aSAH patients, a retrospective, single-institution cohort study found that 58% of patients were maintained within ARDSNet parameters, including tidal volumes of ≤ 8 mL/kg, yet there were no ventilator settings that predicted the development of ARDS [12]. The presence of ARDS risk factors, defined as sepsis, shock, pneumonia, gastric aspiration, and transfusion, were the only findings associated with the development of ARDS. As opposed to other studies, the clinical severity of aSAH did not correlate with the development of ARDS [12]. The development of ARDS was associated with increased duration of intensive care unit (ICU) stay, but not mortality [12].

A prospective, single-center study of 499 patients with acute brain injury, including SAH, found that lower tidal volumes and higher positive end-expiratory pressure (PEEP) resulted in decreased duration of MV from 14.9 to 12.6 days and increased 90-day ICU days [25]. In a multicenter study of all acute brain injury patients including SAH, a protocol of low tidal volume (≤ 7 mL/kg), moderate PEEP (6–8 cmH_2_O), and early extubation was associated with a decrease in mortality and number of invasive ventilation-free days [26].

Alveolar collapse is a key pathophysiologic characteristic of ARDS and results in hypoxemia from intrapulmonary shunt [27]. By using PEEP to open collapsed lung units, alveolar recruitment is one strategy to maintain functional residual capacity and thereby improve oxygenation in ARDS [28, 29]. However, lung recruitment remains controversial, and high PEEP ventilation was associated with higher mortality compared to low PEEP ventilation in a randomized control trial [30]. The applicability of these trials to patients with aSAH is limited because they excluded patients with elevated ICP or acute brain injury.

To address this limitation, several small retrospective clinical studies have attempted to examine the relationship between lung-protective ventilation and ICP. One retrospective, single-institution review reported outcomes for 12 aSAH patients receiving lung-protective ventilation settings with resultant hypercapnia (defined as PaCO_2_ 50–60 mmHg) and found that these patients had no increase in their ICP compared to patients with a PaCO_2_ of 40 mmHg [31]. The authors hypothesized that while pial arteries vasodilate in response to hypercapnia, there is some evidence that the major cerebral arteries and intracortical arteries constrict instead, possibly accounting for the unchanged ICP they observed [31, 32]. A randomized study of lung recruitment methods in aSAH patients with ARDS by Nemer et al. compared different alveolar recruitment maneuvers [33]. One arm was subjected to 35 cmH_2_0 of continuous positive airway pressure for 40 s, termed continuous positive airway pressure recruitment maneuver (CRM), while the other underwent a pressure control recruitment maneuver (PCRM) of a PEEP of 15 cmH_2_0 with pressure control above PEEP of 35 cmH_2_0 for 2 min [33]. Compared to baseline, they found CRM to be associated with a higher ICP (20.50 ± 4.75 vs 13.13 ± 3.56 mmHg) and a lower cerebral perfusion pressure (CPP) (62.38 ± 9.81 vs 79.60 ± 6.8 mmHg) with no significant improvement in PaO_2_:FiO_2_ ratio (110.9 ± 24.7 to 112.6 ± 26.7). PCRM on the other hand had no significant effect on ICP but increased CPP (84.25 ± 5.48 to 79 ± 6.80 mmHg) [33]. PCRM was also associated with a clinically significant improvement in PaO_2_:FiO_2_ ratio (108.5 to 203.6) [33].

One prospective, single-institution observational study evaluated the longitudinal effect of PEEP on ICP in aSAH patients [34]. The authors found that, compared to a group with PEEP of 5 cmH_2_O, patients with a PEEP of 20 cmH2O had no significant effect on ICP on post-bleed days 1 and 3, but did experience significantly higher ICP on post-bleed day 7 (19.5 vs 11 mmHg). Post-bleed day 7 is an important milestone in the natural history of aSAH because maximal vasospasm can occur between days 6 and 8 [35]. Severe vasospasm may lead to reduced CBF and cause cerebral ischemia and edema. The elevated PEEP group also experienced a decrease in mean arterial pressure (MAP) from baseline and subsequently a decrease in cerebral blood flow, thought to be a result of ineffective cerebral autoregulation [34]. The authors postulated that cerebral edema, in conjunction with elevated intracranial venous pressure and diminished intracranial venous outflow due to elevated PEEP, led to increased ICP [34].

A retrospective, single-institution review of patients with severe neurologic injuries (GCS < 9, 37.5% with aSAH) who required MV and ICP monitoring found no significant association between PEEP and ICP or CPP, except in patients with severe lung injury (PaO_2_/FiO_2_ < 100) [36]. On multivariate analysis of severe lung injury patients, every 1-cmH_2_O increase in PEEP was associated with a 0.31-mmHg increase in ICP (*p* = 0.04) and a 0.85-mmHg decrease in CPP (*p* = 0.02) [36]. The study did not report any subgroup analysis of the various pathologies included in their cohort or investigate if the mode of MV used had an effect on their results. On the other hand, a prospective study of 21 comatose patients with normal lung compliance and abnormal lung compliance were subjected to increases in PEEP while measuring central venous pressure (CVP), CPP, ICP, cerebral compliance, and mean middle cerebral artery velocity [37]. In those with normal lung compliance, PEEP increases caused an increase in CVP but reduced MAP, CPP, and mean velocities while ICP and cerebral compliances stayed the same. In those with low compliance, there was no variation in any of the variables with increases in PEEP.

#### Recommendations

In summary, the literature suggests that an increase in PEEP decreases MAP and increases intracranial pressure. However, since ARDS can present early in these patients, higher PEEPs may be safe early in the course of a patient with aSAH without evidence of intracranial hypertension or mass effect from hematoma. It is reasonable to use ICPM with ability of CSF diversion as the patient approaches the peak of the DCI period, prior to increasing PEEP to treat lung pathology.

### Prone positioning

Prone position ventilation improves gas exchange in patients with ARDS and other pathologic states with ventilation-perfusion mismatch such as NPE. One retrospective study of sixteen patients with aSAH, HH grade III or higher with ICPM described proning [38]. With proning, there was a significant increase in PaO_2_ (from 97.3 ± 20.7 to 126.6 ± 31.7 Torr) and PbtO_2_ (from 26.8 ± 10.9 to 31.6 ± 12.2 Torr) along with ICP (from 9.3 ± 5.2 to 14.8 ± 6.7 mmHg) while CPP decreased (from 73.0 ± 10.5 to 67.7 ± 10.7 mmHg) [38]. In a retrospective review of 29 patients with ICPM and acute brain injury, the mean baseline ICP in a supine position was 9.5 ± 5.9 mmHg which increased significantly during prone positioning to 15.4 ± 6.2 mmHg [39]. They found no significant difference between CPP in a supine position (82 ± 14.5 mmHg) or a prone position (80.1 ± 14.1 mmHg) [39]. Another prospective study of proning in 8 patients with TBI and SAH found similar results as the prior study with a statistically significant increase in PaO_2_ (from 12.6 ± 1.4 to 15.7 ± 3.2 kPa) and ICP (from 12 ± 6 to 15 ± 4 mmHg) however with improvement in CPP (from 66 ± 7 to 73 ± 8 mmHg) [40]. MAP improved in these patients (from 78 ± 8 to 88 ± 8 mmHg) [40]. The authors postulate better venous return in the prone position improved MAP to a greater extent than ICP, resulting in improved CPP [40]. Finally, another prospective trial in 11 patients with TBI and SAH found that proning had no significant effect on ICP, CPP, or MAP but significantly increased PaO_2_ (from 13.2 ± 2.1 to 19.1 ± 6.1 kPa) [41]. The authors comment on increasing sedation on a patient who had an immediate increase in ICP on proning which highlights that results in non-controlled studies may be confounded. It is important to note neither of these studies had patients with ARDS.

#### Recommendations

Based on these studies, proning can be expected to raise ICP significantly however dramatically improves oxygenation. Patients with aSAH with ICPM who have demonstrated stable ICPs, have no mass effect from intracranial hematoma or edema, and who are experiencing ARDS can be considered for proning.

### Alternative modes of ventilation

Airway pressure release ventilation (APRV), a pressure-limited, time-cycled mode of MV that allows spontaneous breathing, is another treatment modality in the management of ARDS. APRV utilizes inverse ratio ventilation (IRV), whereby the inspiratory time is longer than the expiratory time. This increases alveolar recruitment and improves oxygenation [42].

Our search yielded one single case report describing the use of APRV in aSAH, resulting in improvement in oxygenation, alveolar ventilation, and cerebral blood flow with a negligible increase in ICP [43]. One study that examined IRV in a rabbit aSAH model compared to normal ratio ventilation did not find CPP to be significantly different, but did find significantly elevated mean airway pressures and slightly elevated ICP above baseline [44]. In another study, 22 Yorkshire swine undergoing controlled lung injury to mimic ARDS and intracranial pathology with ICP elevation to 30–40 with intracranial balloon were randomized to ARDSNet, APRV, or sham, and blood gases, quantitative histopathology, and cerebral microdialysis were assessed [45]. The investigators found no difference in FiO_2_, CVP, end-tidal CO_2_, MAP, CPP, and ICP between the groups, but statistically improved P/F ratio and higher mean airway pressures in the APRV group [45]. They also found no differences in arterial pH, PaCO_2_, PaO_2_, and SaO_2_ at the common carotid or venous pH, lactate, SvO_2_, or PvO_2_ at femoral and jugular sites with the only difference of APRV having lower PvCO_2_ at the jugular site [45]. Cerebral dialysis showed lower lactate in the APRV group but lactate pyruvate ratios insignificantly different [45]. The two main limitations of this study are the dropout bias due to death of six animals not included and analysis for only 6.5 h.

#### Recommendations

Consider APRV in this population if there is concern for vent asynchrony or ARDS but need to maintain ICP.

## Delayed cerebral ischemia

### Ventilation

DCI is one of the most dreaded complications of aSAH and is a significant contributor to long-term morbidity [46]. Angiographic vasospasm can be seen independently or in conjunction with DCI in patients with aSAH [35]. There is significant heterogeneity in the literature among definitions and endpoints in studies describing the phenomenon of post-aSAH ischemia, with some studies using vasospasm as a surrogate marker or interchangeably with DCI [46]. We found existing data describing DCI, vasospasm, and MV to be contradictory, possibly due to this heterogeneity. One retrospective, single-institution review found DCI to be independently associated with prolonged MV (HR 1.61; 95% CI 1.02–2.56) [47]. A relationship between pulmonary complications and DCI was echoed by a second retrospective, single-institution study, which determined pneumonia was independently associated with the development of DCI (adjusted OR, 2.0; 95% CI 1.1–3.7) [48]. An understanding of this relationship is not firmly established, however. A retrospective, single-institution cohort study found the development of ARDS did not appear to increase the risk of vasospasm [11]. One prospective, observational study examined the effect of cerebral vasospasm on CBF in the setting of elevated PEEP, finding that vasospasm did not appear to influence MAP or CBF at PEEP up to 20 cmH20 [34]. However, increasing PEEP resulted in codependent decreases in MAP and CBF in patients regardless of the presence of vasospasm. Additionally, MAP and CBF responded as expected to medical therapy in both populations [49]. This suggests that application of PEEP is no worse for patients with vasospasm than without, though this cannot necessarily be applied to patients with DCI, and further studies are needed to elucidate this.

### Recommendations

In summary, one of the most challenging scenarios in managing high-grade aSAH patients is preventing the occurrence of DCI while there is concurrent severe ARDs. Higher PEEPs may decrease CBF in this group of patients; thus, simultaneous use of ionotropic agents such as milrinone may offset the decreased venous return from higher PEEPs. These patients are typically ideal candidates for advanced intracranial monitoring with PbtO_2_, potentially allowing for more nuanced ventilator titration.

## Conclusions

Overall, there is a paucity of high-level data on the effects of MV on patients with aSAH, with the result that no definitive management statements can be made. However, we can summarize the data along with our experience with the following suggestions:

1. Hypercapnia may be effective in reducing DCI and improving outcomes, but these patients should have some form of ICPM if higher PaCO_2_ will be targeted.
2. ARD protocol ventilation should be followed in patients with aSAH allowing for higher PEEPs if early in the bleed course and after aneurysm has been secured. These patients should have ICPM, especially during the DCI period, and advance intracranial monitoring can help guide ventilator titration to strike a balance between oxygenation, ventilation, PEEP, and cerebral perfusion.
3. Spontaneous modes of ventilation such as APRV may be considered in patients with concomitant ARDs and in the DCI period. This may lower sedation requirements, which, in addition to ionotropic agents, may meet goals of cerebral perfusion. Hypercapnia during this period as discussed above may be beneficial.

## Data Availability

Not applicable

## Abbreviations

APRV: Airway pressure release ventilation
ARDS: Acute respiratory distress syndrome
aSAH: Aneurysmal subarachnoid hemorrhage
CBF: Cerebral blood flow
CSF: Cerebrospinal fluid
CO_2_: Carbon dioxide
CPP: Cerebral perfusion pressure
CRM: Continuous positive airway pressure recruitment maneuver
CVP: Central venous pressure
DCI: Delayed cerebral ischemia
EVD: External ventricular drain
FG: Fisher grade
FiO_2_: Fraction of inspired oxygen
HH: Hunt Hess
ICP: Intracranial pressure
ICPM: Intracranial pressure monitoring
ICU: Intensive care unit
IRV: Inverse ratio ventilation
MAP: Mean arterial pressure
MV: Mechanical ventilation
NPE: Neurogenic pulmonary edema
O_2_: Oxygen
PaCO_2_: Partial pressure of CO_2_
PbtO_2_: Brain partial oxygen pressure
PCRM: Pressure control recruitment maneuver
PEEP: Positive end-expiratory pressure
VAP: Ventilator-acquired pneumonia
